# Efficacy and safety of GLP-1 receptor agonists as add-on to SGLT2 inhibitors in type 2 diabetes mellitus: A meta-analysis

**DOI:** 10.1038/s41598-019-55524-w

**Published:** 2019-12-18

**Authors:** Marco Castellana, Angelo Cignarelli, Francesco Brescia, Sebastio Perrini, Annalisa Natalicchio, Luigi Laviola, Francesco Giorgino

**Affiliations:** Section of Internal Medicine, Endocrinology, Andrology and Metabolic Diseases, Department of Emergency and Organ Transplantation, University of Bari Aldo Moro, Bari, Italy

**Keywords:** Type 2 diabetes, Type 2 diabetes

## Abstract

GLP-1 receptor agonists (GLP-1RA) and SGLT2 inhibitors (SGLT2i) have been associated with improved glycemic control, body weight loss and favorable changes in cardiovascular risk factors and outcomes. We conducted a systematic review and meta-analysis to evaluate the effects of the addition of GLP-1RA to SGLT2i in patients with type 2 diabetes mellitus and inadequate glycemic control. Six databases were searched until March 2019. Randomized controlled trials (RCT) with a follow-up of at least 24 weeks reporting on HbA1c, body weight, systolic blood pressure, lipids, achievement of HbA1c < 7%, requirement of rescue therapy due to hyperglycemia and hypoglycemic events were selected. Four RCTs were included. Compared to SGLT2i, the GLP-1RA/SGLT2i combination was associated with greater reduction in HbA1c (−0.74%), body weight (−1.61 kg), and systolic blood pressure (−3.32 mmHg). A higher number of patients achieved HbA1c < 7% (RR = 2.15), with a lower requirement of rescue therapy (RR = 0.37) and similar incidence of hypoglycemia. Reductions in total and LDL cholesterol were found. The present review supports treatment intensification with GLP-1RA in uncontrolled type 2 diabetes on SGLT2i. This drug regimen could provide improved HbA1c control, together with enhanced weight loss and blood pressure and lipids control.

## Introduction

Diabetes mellitus is a chronic disease characterized by high prevalence, morbidity and excess mortality. It is a leading cause of cardiovascular disease, end-stage renal disease and blindness, causing a relevant economic impact on patients, their families and the health care system^1^. To reduce the incidence and progression of these complications, particularly microvascular, glycemic management aiming at blood glucose concentrations close to the normal range has been proved effective^2^. Management of hyperglycemia and other cardiovascular risk factors should be thus actively pursued, and combination therapies should be attentively considered in individuals with inadequate metabolic control^3^.

In the last 10 years, two new drug classes have been available for type 2 diabetes therapy, GLP-1 receptor agonists (GLP-1RA) and SGLT-2 inhibitors (SGLT2i). GLP-1RA can be classified into short-acting (exenatide, lixisenatide) and long-acting (albiglutide, dulaglutide, exenatide long-acting release, liraglutide, semaglutide), based on their pharmacokinetic and pharmacodynamic profile. These agents stimulate insulin release in a glucose-dependent manner, promote reduction in glucagon secretion and hepatic glucose production, slow gastric emptying, and suppress appetite^4–7^. The most used SGLT2i include canagliflozin, dapagliflozin and empagliflozin. They inhibit glucose reabsorption by the kidney, thus increasing its excretion in the urine and ameliorating the effects of glucotoxicity on beta-cells; however, they increase glucagon levels. Both classes promote weight loss and blood pressure lowering, albeit with different and complementary mechanisms, and are characterized by a low risk of hypoglycemia^8^. Moreover, some of the agents in these drug classes have also been associated with reduction in cardiovascular events and mortality and nephroprotection^9–13^.

Recently, a consensus report by the American Diabetes Association and the European Association for the Study of Diabetes on treatment of hyperglycemia in type 2 diabetes was released. In patients with established atherosclerotic cardiovascular disease or chronic kidney disease already taking SGLT2i, a combination of GLP-1RA and SGLT2i should be considered if further intensification of glycemic control is required^14^. The GLP-1RA/SGLT2i combination should be also preferentially used over other therapies in inadequately controlled patients in which promoting weight loss is a priority^14^. Considering their specific mechanistic synergy, tackling multiple pathophysiological defects of type 2 diabetes, the combination of GLP-1RA and SGLT-2i is expected to result in further decrease in HbA_1c_ with no further risk of hypoglycaemia, greater weight loss, and enhanced potential for cardiovascular and renal benefits, as compared with either drug class alone. Since studies evaluating the effects of the addition of GLP-1RA to SGLT2i in patients with inadequately controlled type 2 diabetes are now available, we performed a systematic review and meta-analysis focusing on traditional glycemic targets as well as on other major risk factors for cardiovascular disease, including hypertension, obesity, and dyslipidemia. Specifically, a comparison of the effects of the GLP-1RA/SGLT2i combination versus SGLT2i on HbA1c, body weight, systolic blood pressure (SBP), lipids, achievement of HbA1c < 7%, requirement of rescue therapy due to hyperglycemia, and incidence of hypoglycemic events was carried out.

## Materials and Methods

The systematic review was registered in PROSPERO (CRD42018110532) and performed in accordance with the Preferred Reporting Items for Systematic Reviews and Meta-Analyses (PRISMA) statement (Supplementary Appendix)^15^.

### Search strategy

A four-step search strategy was planned. First, we identified keywords and MeSH terms in PubMed. Second, the terms “glucagon-like peptide-1 receptor agonist” and “sodium glucose cotransporter 2 inhibitor” (including exenatide, lixisenatide, albiglutide, dulaglutide, liraglutide, semaglutide, taspoglutide, canagliflozin, dapagliflozin, empagliflozin, ertugliflozin, ipragliflozin) were searched in PubMed, CENTRAL, ClinicalTrials.gov, EudraCT, Scopus and Web of Science. Third, randomized controlled trials (RCT) with a follow-up of at least 24 weeks analyzing GLP-1RA as add-on to SGLT2i in type 2 diabetes mellitus were selected. Fourth, references of included studies were searched for additional papers. The last search was performed on March 5^th^, 2019. No language restriction was adopted. Two investigators (MC, FG) independently searched papers, screened titles and abstracts of the retrieved articles, reviewed the full-texts, and selected articles for their inclusion.

### Data extraction

The following information was extracted independently by the same investigators in a piloted form: 1) general information on the study (author, year of publication, study name, study type, follow-up period, number of patients, age, diabetes duration, ethnicity, sex, inclusion criteria of screened population, glucose-lowering medications at pre-screening, treatment of randomization, other anti-diabetes therapies allowed during the study); 2) end-points, including HbA1c, body weight, SBP, lipids, number of patients achieving an HbA1c target of less than 7%, number of patients requiring rescue therapy due to hyperglycemia, incidence of hypoglycemic events. The criteria for requirement of rescue therapy due to hyperglycemia and the definition of hypoglycemia for each study can be found in the Supplementary Appendix. The main paper and supplementary data were searched; if data was missing, the study protocol and pharmaceutical industry website were searched. Data were cross-checked, and any discrepancy was discussed.

### Study quality assessment

The risk of bias of included studies was assessed independently by two reviewers (MC, FG) through the Cochrane Collaboration’s tool for assessing risk of bias for the following aspects: random sequence generation; allocation concealment; blinding of participants and personnel; blinding of outcome assessment; incomplete outcome data; selecting reporting. For other bias, funding and authorship were assessed. Each domain was assigned low, unclear or high risk of bias^16^.

### Data analysis

The primary outcome was the change in HbA1c from baseline to the last available follow-up. Secondary outcomes included changes in body weight, SBP and lipids from baseline to the last available follow-up, achievement of an HbA1c target of less than 7%, requirement of rescue therapy due to hyperglycemia, incidence of hypoglycemic events. The first four endpoints were analyzed as continuous variables and summarized as weighted mean difference; the last three as dichotomous, and the risk ratios (RR) were estimated. If standard deviation was missing in a study for a specific outcome, it was calculated from standard error, 95% confidence interval or from interquartile range; if none of these were available, the largest among the other studies was reported. For studies with three arms, the shared one was used for comparison of the other two; this shared group was split into two groups with smaller sample size, and two comparisons were included. Heterogeneity between studies was assessed by using I^2^, with 50% or higher regarded as high. Publication bias was assessed with Egger’s test; the trim-and-fill method was used for estimating its effect. Sensitivity analyses by removing each study in turn were also performed. In particular, a specific sensitivity analysis was carried out to assess the impact of including patients on basal insulin as background therapy. All analyses were two-sided and were carried out using RevMan5.3 (The Cochrane Collaboration) and Prometa3.0 (Internovi) with a random-effect model; p < 0.05 was regarded as significant.

### Ethics

These systematic review and meta-analysis were in accordance with the principles of the Declaration of Helsinki. Analyses were performed on data extracted from published papers.

## Results

### Study characteristics

**Figure 1:**
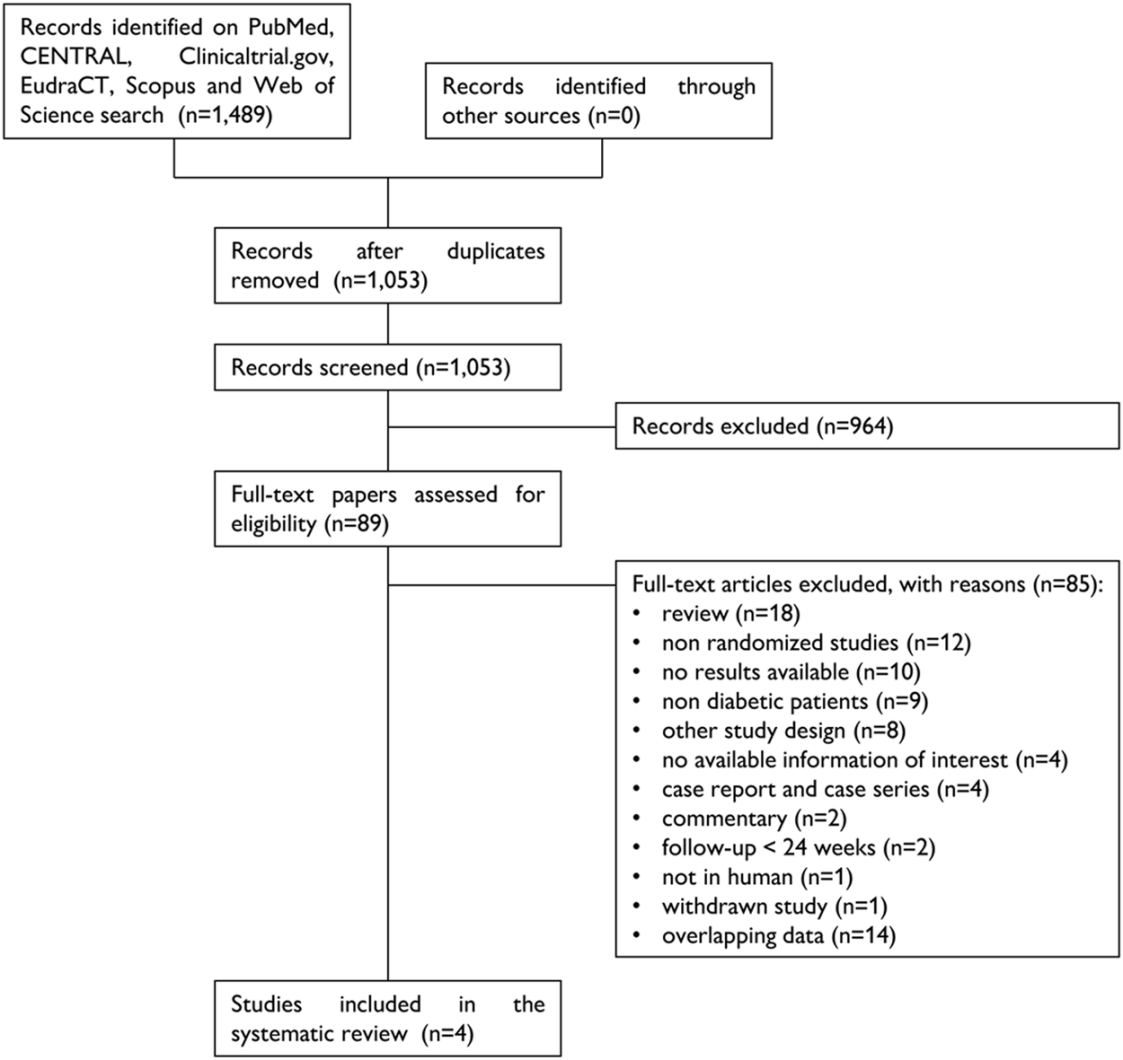
Flow-chart of the systematic review.

A total of 1,489 papers were found, of which 390 on PubMed, 2 on ClinicalTrials.gov, 15 on EudraCT, 94 on CENTRAL, 762 on Scopus and 226 on Web of Science. After removal of 436 duplicates, 1,053 articles were analyzed for title and abstract; 964 records were excluded (systematic reviews, meta-analyses, non-randomized studies, comparison of therapy schemes other than the one reported above, cost-effectiveness studies, studies recruiting patients with type 1 diabetes mellitus, studies not in humans). The remaining 89 papers were retrieved in full-text and four articles were finally included in the systematic review (Fig. 1)^17–20^. No additional study was retrieved after screening the references of these papers.

### Study quality assessment

The risk of bias of the included studies is shown in Supplementary Appendix. Random sequence generation was reported only in AWARD-10^18^. Allocation concealment and selective reporting bias were adequate in all. In DUAL IX, the open-label design led to the assignment of a high risk of bias for blinding of participants and personnel and blinding of outcome assessment: iDegLira and glargine share the route of administration and titration, thus a double-blind design could have been potentially considered^19^. In SUSTAIN 9, premature discontinuation was more frequent in the semaglutide arm compared to placebo, with possible attrition bias^20^. Finally, an industrial sponsor funded the study in all^17–20^.

### Qualitative analysis (systematic review)

The characteristics of the included articles are summarized in Table 1. The studies were published between 2018 and 2019, had sample sizes ranging from 302 to 464 patients, and a follow-up from 24 to 52 weeks. All studies were randomized controlled, multinational and sponsored by industry (one by AstraZeneca, one by Eli Lilly, two by Novo Nordisk). One study examined dulaglutide, one exenatide QW, one iDegLira, and one semaglutide. Two studies were three-armed^17,18^. Participants were adult outpatients diagnosed with type 2 diabetes mellitus, with HbA1c 7–12% and BMI 20–45 kg/m^2^. Regarding the glucose-lowering therapy at pre-screening, patients were on SGLT2i with or without metformin in three trials^18–20^, metformin only in one^17^. 1,610 patients were included, 53% were males, and 85% were Caucasian. The weighted-mean age was 56.3 ± 9.7 years, and the weighted-mean duration of diabetes was 8.7 ± 6.1 years. 876 were randomized to GLP-1RA added to SGLT2i, while 734 to SGLT2i. Moreover, in DURATION-8, 231 patients were randomized to GLP-1RA added to placebo; they were not included in the present review.

**Table 1:** Qualitative analysis of studies included in the systematic review.

Study name (identifier)	Author, year	GLP-1RA + SGLT2i arm	SGLT2i arm	Other therapies	Study type	Follow-up (weeks)	Number of patients	Population	Age (years)	Diabetes duration (years)	HbA1c	Body weight	Systolic blood pressure	Lipids	HbA1c < 7%	Rescue therapy	Hypoglycemic events
AWARD-10 (NCT02597049)	Ludvik, 2018	Dulaglutide QW + SGLT2i	Placebo + SGLT2i	Metformin	RCT	24	424	type 2 diabetes, HbA1c 7–9.5%, BMI ≤ 45 kg/m^2^	57.3 (9.4)	9.4 (6.2)	x	x	x	x	x	x	x
DUAL IX (NCT02773368)	Philis-Tsimikas, 2019	iDegLira QD + SGLT2i	Glargine + SGLT2i	Metformin	RCT	26	420	type 2 diabetes, HbA1c 7–11%, BMI 20–40 kg/m^2^	56.7 (10.3)	9.6 (6.3)	x	x	x	x	x		x
DURATION-8 (NCT02229396)	Jabbour, 2018	Exenatide QW + Dapagliflozin	Placebo + Dapagliflozin	Metformin	RCT	52	464	type 2 diabetes, HbA1c 8–12%	54.5 (9.5)	7.3 (5.7)	x	x	x	x	x	x	
SUSTAIN 9 (NCT03086330)	Zinman, 2019	Semaglutide QW + SGLT2i	Placebo + SGLT2i	Metformin, sulphonylurea	RCT	30	302	type 2 diabetes, HbA1c 7–10%	57 (9.5)	—	x	x	x	x	x	x	x

BMI, body mass index; GLP-1RA, glucagon-like peptide-1 receptor agonist; QD, once daily; QW, once weekly; RCT, randomized controlled trial; SGLT2i, sodium glucose cotransporter 2 inhibitor.

### Quantitative analysis (meta-analysis)

**Figure 2:**
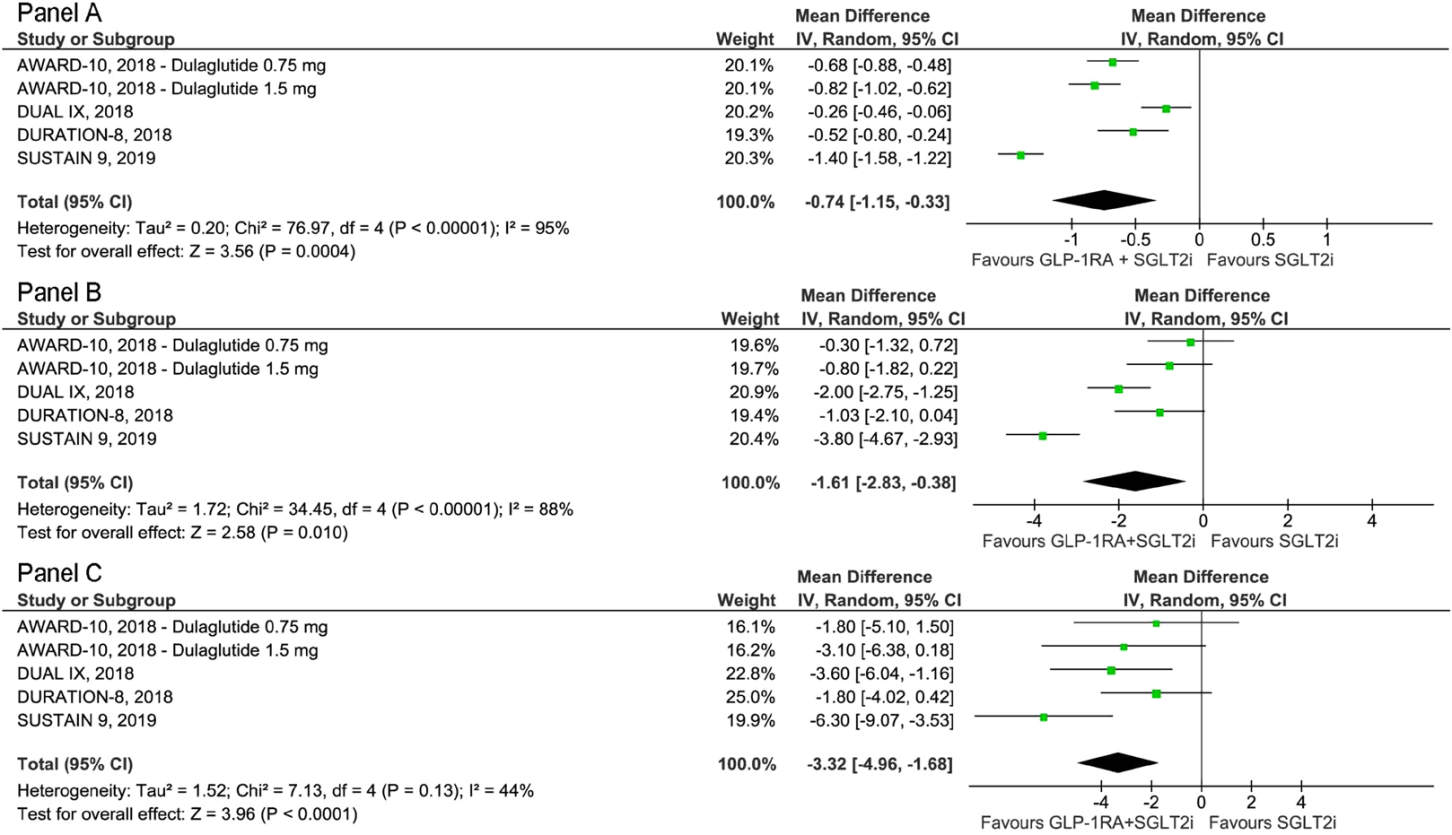
Forest plots of meta-analysis for change in HbA1c (panel A), body weight (panel B), and systolic blood pressure (panel C) from baseline to the last available follow-up.

The primary outcome was the change in HbA1c from baseline to the last available follow-up. The weighted-mean HbA1c at baseline was 8.5% with no difference between arms (p = 0.30). The GLP-1RA/SGLT2i combination was associated with an improved glycemic control, expressed as change in HbA1c, versus SGLT2i (Δ = −0.74%; 95% CI −1.15 to −0.33; p < 0.001; I^2^ = 95%) (Fig. 2, panel A). Moreover, the GLP-1RA/SGLT2i combination showed to be superior to SGLT2i in achieving an HbA1c value < 7% (RR = 2.15; 95% CI 1.20 to 3.86; p = 0.01; I^2^ = 96%), with fewer patients requiring rescue therapy due to hyperglycemia (RR = 0.37; 95% CI 0.15 to 0.89; p = 0.03; I^2^ = 53%) (Supplementary Appendix).

Body weight at baseline was 90.6 kg, with no differences between the study arms (p = 0.48). The GLP-1RA/SGLT2i combination caused a greater body weight loss versus SGLT2i (Δ = −1.61 kg; 95% CI −2.83 to −0.38; p = 0.01; I^2^ = 88%) (Fig. 2, panel B).

SBP at baseline was 129 mmHg, with no differences between the two groups (p = 0.91). The GLP-1RA/SGLT2i combination was also associated with a lowering in SBP versus SGLT2i (Δ = −3.32 mmHg; 95% CI −4.96 to −1.68; p < 0.001; I^2^ = 44%) (Fig. 2, panel C). In regard to incidence of hypoglycemic events, GLP-1RA added to SGLT2i showed a similar incidence as SGLT2i (RR = 1.43; 95% CI 0.46 to 4.52; p = 0.54; I^2^ = 94%). Finally, reductions in total cholesterol (Δ = −0.17 mmol/l; 95% CI −0.32 to −0.02; p = 0.02; I^2^ = 66%) and LDL cholesterol (Δ = −0.13 mmol/l; 95% CI −0.24 to −0.03; p = 0.01; I^2^ = 50%) were observed, with no changes in either HDL cholesterol (p = 0.61) or triglycerides (p = 0.09) (Supplementary Appendix).

We found only one study with basal insulin as background therapy (DUAL IX)^19^. When this study was excluded from the analysis, improvements in HbA1c (Δ = −0.86%; 95% CI −1.25 to −0.47; p < 0.001; I^2^ = 93%), SBP (Δ = −3.25 mmHg; 95% CI −5.43 to −1.06; p = 0.004; I^2^ = 57%), LDL cholesterol (Δ = −0.12 mmol/l; 95% CI −0.25 to −0.00; p = 0.05; I^2^ = 61%) and chance of achieving an HbA1c value < 7% (RR = 2.52; 95% CI 1.78 to 3.58; p < 0.001; I^2^ = 75%) were confirmed. Similarly, there were neutral effects on the incidence of hypoglycemia (p = 0.10), HDL cholesterol (p = 0.40) and triglycerides (p = 0.07), as in the analysis of the full trial set. However, non-significant trends to greater effects on body weight loss (Δ = −1.50 kg; 95% CI −3.16 to 0.17; p = 0.08) and total cholesterol (Δ = −0.18 mmol/l; 95% CI −0.37 to 0.01; p = 0.06) were observed.

There was no evidence of publication bias, except for achievement of HbA1c < 7%, rescue therapy and hypoglycemic events; the statistical significance of these results was not changed following the application of the trim-and-fill method. In sensitivity analyses, the findings for changes in body weight, total and LDL cholesterol and rescue therapy were not always confirmed (Supplementary Appendix).

## Discussion

This systematic review and meta-analysis was performed to identify a high level of evidence on the efficacy of the combination therapy with GLP-1RA and SGLT2i versus SGLT2i in patients with inadequately controlled type 2 diabetes. We found four RCTs, randomizing 1,610 adult patients with HbA1c between 7–12% and BMI between 20–45 kg/m^2^ to either treatment. The GLP-1RA/SGLT2i combinations versus SGLT2i alone was associated with a higher efficacy on HbA1c reduction, achievement of HbA1c < 7%, body weight loss, SBP reduction, and requirement of rescue therapy due to hyperglycemia, with a similar incidence of hypoglycemic events. A significant reduction in total and LDL cholesterol were also observed, with no changes in either HDL cholesterol or triglycerides.

Current guidelines recommend determining, assessing and pursuing regularly an HbA1c goal on an individual basis, given the strong predictive value for diabetes complications. HbA1c depends on the average glycemia in the previous 2–3 months, but it does not inform about postprandial hyperglycemia or hypoglycemia^3^. The use of some glucose-lowering drugs, specifically sulphonylureas and insulin, is largely limited by the risk of hypoglycemia, which is minimized when using GLP-1RA and SGLT2i^21^. Thus, the results of the present meta-analysis, showing that GLP-1RA/SGLT2i combinations are more effective than SGLT2i on HbA1c, while being characterized by a similar risk of hypoglycemia, are of interest.

Among GLP-1RA/SGLT2i combinations, it is noteworthy that a lower-magnitude reduction in HbA1c with a lower risk of hypoglycemia was found in DUAL IX compared to the other studies. Concerning HbA1c, one possible explanation is the trial design, since iDegLira was compared with glargine as add-on to SGLT2i, and both arms followed a treat to target approach; in regard to hypoglycemia incidence, it could be also due to the effects of liraglutide, differences in the insulin dose, as well as in the type of insulin, since lower rates of hypoglycemia have been reported for insulin degludec compared to insulin glargine U-100^19,22,23^. Moreover, in AWARD-10, an HbA1c reduction higher than reported could have been potentially achieved: a −0.54% HbA1c reduction was observed in the placebo arm, which could also represent a carry-over effect of the late introduction of SGLT2i in many patients (i.e., 3–6 months before study entry)^18^.

Overweight and obesity represent common comorbidities in patients with type 2 diabetes mellitus. A weight loss of at least 5–10% is recommended in these patients, since this is usually associated with improvements in glycemic control and need for medications^3^. Both GLP-1RA and SGLT2i have been associated with weight loss, and one of them, received the approval for the treatment of obesity (i.e. liraglutide)^24,25^. Data from the SCALE Diabetes RCT showed a mean weight loss of 6.4 kg with liraglutide 3.0 mg once-daily and 5.0 kg with liraglutide 1.8 mg/die versus 2.2 kg in the placebo arm in overweight or obese patients with type 2 diabetes followed for 56 weeks; on the other hand, in SCALE Obesity and Prediabetes, a mean weight loss of 8.4 kg on liraglutide 3.0 mg once-daily versus 2.4 kg on placebo among obese patients without type 2 diabetes followed for 56 weeks was reported^26,27^. The above results suggest the difficulty to obtain weight loss with drugs recommended to treat obesity in patients with type 2 diabetes and at lower dosage^28^. This meta-analysis confirms that GLP-1RA induce a further −1.6 kg body weight reduction when added to SGLT2i in individuals with type 2 diabetes. It is worth noting that in SUSTAIN 9, baseline body weight was higher in the SGLT2i arm compared to GLP-1RA/SGLT2i arm; this could have led to an underestimation of treatment difference^20^. Also, in DURATION-8, a significantly higher proportion of participants lost at least 5% of body weight when treated with the combination of exenatide QW and dapagliflozin as compared to either exenatide or dapagliflozin (30.7%, 14.1%, and 21.3%, respectively)^17^. A proposed mechanism for the interaction is the suppression of appetite caused by GLP-1RA, limiting the increased food intake reported to occur with SGLT2i use, in addition to the SGLT2i-mediated glycosuria and consequent calorie loss^10^. Thus, when managing a type 2 diabetic patient with overweight or obesity and inadequately controlled HbA1c, the GLP-1RA/SGLT2i combination can be particularly useful in achieving both glycemic and body weight targets. Of note, in DUAL IX, the intervention and control groups differed in type and dose of basal insulin (e.g. degludec vs glargine), as already stated. Current evidence does not support any difference in change in body weight between these two insulins, therefore the overall results of our meta-analysis evaluating changes in body weight following the addition of GLP-1RA to SGLT2i should not be affected by the trial design and data of DUAL IX^29^.

Hypertension and dyslipidemia represent frequent comorbidities in type 2 diabetes. Although not approved for, both GLP-1RA and SGLT2i have been shown to ameliorate hypertension, and GLP-1RA to improve dyslipidemia^25,30–32^. Therefore, an additive effect when they are used in combination is plausible and it is confirmed by this meta-analysis, in line with available data from other papers^25,33^. Noteworthy, the potential beneficial effect of GLP-1RA on LDL cholesterol may be nullified by the small increase observed with SGLT2i^25,34^. In regard to triglycerides and HDL cholesterol, a small impact has been reported for both classes. Indeed, since the combination of GLP-1RA/SGLT2i results in prominent reductions in HbA1c and body weight, a greater than observed effect on both triglycerides and HDL cholesterol was to be expected^35^.

In DURATION-8, 695 patients were randomized to receive exenatide QW plus dapagliflozin (n = 231), exenatide QW (n = 231), or dapagliflozin (n = 233). This study allows to examine the interaction between the two drug classes on several endpoints. A less than additive effect was found on HbA1c, which could be explained by two hypotheses. As suggested by Polidori *et al*., a subadditive efficacy is expected in combination therapies because of different effective HbA1c levels at baseline on which each drug acts when given as component of a combination strategy, as compared with monotherapy^36^. According to other Authors, SGLT2i cause a rise in hepatic glucose production, which partially offsets the benefits of glycosuria; with increasing HbA1c levels, GLP-1RA may not be able to suppress gluconeogenesis and/or glycogenolysis induced by factors other than glucagon *per se*^37^. Whatever the mechanism, 38%, 30% and 16% of patients achieved an HbA1c < 7% in each arm, respectively. On the other hand, additive effects on body weight and SBP reductions were found, suggesting a mechanistic synergy between the two classes, as already noted^17^.

In October 2019, a meta-analysis on SGLT2i and incretin-based agents combination therapy versus SGLT2i in patients with type 2 diabetes was published by Zhou *et al*.^38^. Three studies on the combination therapy with GLP-1RA and SGLT2i versus SGLT2i were included^17,18,20^. The Author concluded that the former was associated with a higher efficacy on HbA1c (Δ = −0.80%; 95% CI −1.14 to −0.45), body weight (Δ = −1.46 kg; 95% CI −2.38 to −0.54), and SBP (Δ = −2.88 mmHg; 95% CI −4.52 to −1.25); a higher risk of gastrointestinal disorders and similar risk of genital infection, urinary tract infection and hypoglycemia were reported^38^. Despite the statement on the inclusion of the longest follow-up data to avoid duplicating results, data on both changes at week 28 at week 52, respectively, were reported for DURATION-8^38^. Also, no analysis was performed on other outcomes, including change in lipids from baseline to the last available follow-up, achievement of an HbA1c target of less than 7%, and requirement of rescue therapy due to hyperglycemia. Overall, the results of our meta-analysis are consistent with the data above, but evidence was gathered based on a greater number of patients and expanded on additional outcomes.

Several limitations of the present analysis should be discussed. The first limitation ascribes to its aims. Since data on adverse events other than hypoglycemia were not extracted, a full description of the benefits and limits of the addition of GLP-1RA to SGLT2i could not be performed. However, the proportion of patients experiencing treatment-emergent adverse events on GLP-1RA as add-on to SGL2i was similar to GLP-1RA only and in line with current literature, with most of them being of mild-to-moderate intensity^39,40^. Only four RCTs were found, and this is a second limitation. At least four ongoing studies were found, with a sample sizes ranging from 17 to 120 patients and a follow-up from 6 to 32 weeks (Table 2). Also, the results of a fifth study were recently published: in PIONEER-4, 183 patients on SGLT2i at baseline were randomized to oral semaglutide or liraglutide or placebo for 52 weeks. These patients could have potentially been included in our meta-analysis; however, data could not be retrieved even after contacting the corresponding Author of that study^41^. The results of the present review are thus meant to be exploratory; however, they will hardly change following the publication of the results of studies above. Thirdly, we found a high heterogeneity, so caution should be taken in generalizing the results to clinical practice. Specific properties of GLP-1RA and SGLT2i in each trial, study design, or patients’ characteristics other than the extracted ones could explain the finding above. The duration of treatment with SGLT2i was different, since in AWARD-10 it was between 3 and 6 months for the majority of patients, compared to 11 months in SUSTAIN 9^18,20^. Also, we found only one study including patients on basal insulin as background therapy^19^. Fourthly, we were not able to assess the efficacy and safety of different doses of GLP-1RA, either as single drugs (e.g. liraglutide 1.2 mg versus liraglutide 1.8 mg once-daily) or according to the background therapy. Greater effects are to be expected when the highest available doses of GLP-1RA are prescribed to patients on SGLT2i. Lastly, this review included studies on type 2 diabetic patients with a baseline HbA1c ranging from 7 to 12% (in some cases with maximum HbA1c of 9.5%), and a follow-up to up to 52 weeks. Whether including subjects with higher HbA1c levels or a longer follow-up would have led to the same results is still to be assessed.

**Table 2:** Ongoing randomized controlled trials assessing GLP-1RA as add-on to SGLT2i.

Study name (identifier)	GLP-1RA + SGLT2i arm	SGLT2i arm	Study type	Follow-up (weeks)	Number of patients	Population	Status
DECADE (2017-004709-42)	Exenatide QW + Dapagliflozin	Dapagliflozin	RCT	6	17	type 2 diabetes, HbA1c 7.5–10%	Ongoing
DECREASE (NCT03361098)	Exenatide BID + Dapagliflozin	Placebo + Dapagliflozin	RCT	16	64	type 2 diabetes, HbA1c 7–10%, BMI 30–40 kg/m^2^	Recruiting
EXENDA (NCT03007329)	Exenatide QW + Dapagliflozin	Placebo + Dapagliflozin	RCT	24	90	type 2 diabetes, HbA1c 6.5–11%, BMI ≥ 25 kg/m^2^	Recruiting
RESILIENT (2015-005242-60)	Exenatide QW + Dapagliflozin	Placebo + Dapagliflozin	RCT	32	120	type 2 diabetes, HbA1c 6.5–11%, BMI ≥ 30 kg/m^2^	Ongoing

BMI, body mass index; BID, bis in die; GLP-1RA, glucagon-like peptide-1 receptor agonist; QW, once weekly; RCT, randomized controlled trial.

In conclusion, in patients with inadequately controlled type 2 diabetes mellitus, the addition of GLP-1RA to SGLT2i proved to be effective on HbA1c, body weight, SBP, and lipid profile. The chance of achieving HbA1c < 7% is increased, with no further risk of hypoglycemia. Current guidelines, trials results and findings of the present meta-analysis strongly support the GLP-1RA/SGLT2i combination as a strategic option in the management of patients with type 2 diabetes.

## Supplementary information

Supplementary Appendix

## Data Availability

The datasets generated during and/or analysed during the current study are available from the corresponding author on reasonable request.
