# Supplementary material for: Efficacy and safety of GLP-1 receptor agonists as add-on to SGLT2 inhibitors in type 2 diabetes mellitus: A meta-analysis
Source: Sci Rep. 2019 Dec 18;9:19351. doi: 10.1038/s41598-019-55524-w (PMC6920368; doi:10.1038/s41598-019-55524-w)
Supplement: Supplementary file 1 — Supplementary Appendix [file 41598_2019_55524_MOESM1_ESM.pdf]

Efficacy and safety of GLP-1 receptor agonists as add-on to SGLT2 inhibitors in type 2 diabetes mellitus. A meta-analysis.

Marco Castellana[1], Angelo Cignarelli[1], Francesco Brescia[1], Sebastio Perrini[1], Annalisa Natalicchio[1], Luigi Laviola[1], Francesco Giorgino[1].

[1] Section of Internal Medicine, Endocrinology, Andrology and Metabolic Diseases, Department of Emergency and Organ Transplantation, University of Bari Aldo Moro, Bari, Italy.

Supplementary Appendix

1. PRISMA Checklist
2. Table A.1. Criteria for rescue therapy due to hyperglycemia.
3. Table A.2. Definition of hypoglycemia.
4. Figure A.1. Risk of bias graph: review of authors' judgements about each risk of bias item presented as percentages across all included studies.
5. Figure A.2. Risk of bias summary: review of authors' judgements about each risk of bias item for each included study.
6. Figure A.3. Forest plot of meta-analysis for prevalence of patients achieving HbA1c < 7%.
7. Figure A.4. Forest plot of meta-analysis for number of patients requiring rescue therapy due to hyperglycemia.
8. Figure A.5. Forest plot of meta-analysis for incidence of hypoglycemia.
9. Figure A.6. Forest plot of meta-analysis for change in Total Cholesterol from baseline to the last available follow-up.
10. Figure A.7. Forest plot of meta-analysis for change in HDL Cholesterol from baseline to the last available follow-up.
11. Figure A.8. Forest plot of meta-analysis for change in LDL Cholesterol from baseline to the last available follow-up.
12. Figure A.9. Forest plot of meta-analysis for change in Triglycerides from baseline to the last available follow-up.
13. Table A.3. Sensitivity analysis and publication bias.

1. PRISMA Checklist

Section/topic	#	Checklist item	Reported on page #
**TITLE**			
Title	1	Identify the report as a systematic review, meta-analysis, or both.	1
**ABSTRACT**			
Structured summary	2	Provide a structured summary including, as applicable: background; objectives; data sources; study eligibility criteria, participants, and interventions; study appraisal and synthesis methods; results; limitations; conclusions and implications of key findings; systematic review registration number.	2
**INTRODUCTION**			
Rationale	3	Describe the rationale for the review in the context of what is already known.	3-4
Objectives	4	Provide an explicit statement of questions being addressed with reference to participants, interventions, comparisons, outcomes, and study design (PICOS).	3-4
**METHODS**			
Protocol and registration	5	Indicate if a review protocol exists, if and where it can be accessed (e.g., Web address), and, if available, provide registration information including registration number.	4
Eligibility criteria	6	Specify study characteristics (e.g., PICOS, length of follow-up) and report characteristics (e.g., years considered, language, publication status) used as criteria for eligibility, giving rationale.	4
Information sources	7	Describe all information sources (e.g., databases with dates of coverage, contact with study authors to identify additional studies) in the search and date last searched.	4
Search	8	Present full electronic search strategy for at least one database, including any limits used, such that it could be repeated.	4
Study selection	9	State the process for selecting studies (i.e., screening, eligibility, included in systematic review, and, if applicable, included in the meta-analysis).	4
Data collection process	10	Describe method of data extraction from reports (e.g., piloted forms, independently, in duplicate) and any processes for obtaining and confirming data from investigators.	4
Data items	11	List and define all variables for which data were sought (e.g., PICOS, funding sources) and any assumptions and simplifications made.	4
Risk of bias in individual studies	12	Describe methods used for assessing risk of bias of individual studies (including specification of whether this was done at the study or outcome level), and how this information is to be used in any data synthesis.	5
Summary measures	13	State the principal summary measures (e.g., risk ratio, difference in means).	5
Synthesis of results	14	Describe the methods of handling data and combining results of studies, if done, including measures of consistency (e.g., I^2) for each meta-analysis.	5

Section/topic	#	Checklist item	Reported on page #
Risk of bias across studies	15	Specify any assessment of risk of bias that may affect the cumulative evidence (e.g., publication bias, selective reporting within studies).	5
Additional analyses	16	Describe methods of additional analyses (e.g., sensitivity or subgroup analyses, meta-regression), if done, indicating which were pre-specified.	5
**RESULTS**			
Study selection	17	Give numbers of studies screened, assessed for eligibility, and included in the review, with reasons for exclusions at each stage, ideally with a flow diagram.	5
Study characteristics	18	For each study, present characteristics for which data were extracted (e.g., study size, PICOS, follow-up period) and provide the citations.	6
Risk of bias within studies	19	Present data on risk of bias of each study and, if available, any outcome level assessment (see item 12).	6
Results of individual studies	20	For all outcomes considered (benefits or harms), present, for each study: (a) simple summary data for each intervention group (b) effect estimates and confidence intervals, ideally with a forest plot.	6
Synthesis of results	21	Present results of each meta-analysis done, including confidence intervals and measures of consistency.	6-7
Risk of bias across studies	22	Present results of any assessment of risk of bias across studies (see Item 15).	6
Additional analysis	23	Give results of additional analyses, if done (e.g., sensitivity or subgroup analyses, meta-regression [see Item 16]).	6-7
**DISCUSSION**			
Summary of evidence	24	Summarize the main findings including the strength of evidence for each main outcome; consider their relevance to key groups (e.g., healthcare providers, users, and policy makers).	7-8
Limitations	25	Discuss limitations at study and outcome level (e.g., risk of bias), and at review-level (e.g., incomplete retrieval of identified research, reporting bias).	10
Conclusions	26	Provide a general interpretation of the results in the context of other evidence, and implications for future research.	11
**FUNDING**			
Funding	27	Describe sources of funding for the systematic review and other support (e.g., supply of data); role of funders for the systematic review.	16

From: Moher D, Liberati A, Tetzlaff J, Altman DG, The PRISMA Group (2009). Preferred Reporting Items for Systematic Reviews and Meta-Analyses: The PRISMA Statement. PLoS Med 6(7): e1000097. doi:10.1371/journal.pmed1000097

For more information, visit: **www.prisma-statement.org**.

Table A.1. Criteria for rescue therapy due to hyperglycemia.

Study name (identifier)	Criteria
AWARD-10 (NCT02597049)	- average daily plasma glucose from the four-point self-monitoring of blood glucose profile >240 mg/dl over at least a two-week period any time during the first 4 weeks post-randomization<br>- average daily plasma glucose >200 mg/dl over a two-week period at any time after the first four weeks post-randomization
DUAL IX (NCT02773368)	Not applicable
DURATION-8 (NCT02229396)	- fasting plasma glucose more than 270 mg/dl between weeks 8 and 12<br>- fasting plasma glucose more than 240 mg/dl between weeks 12 and 20<br>- fasting plasma glucose more than 200 mg/dl between week 20 and study end
SUSTAIN 9 (NCT03086330)	- any fasting plasma glucose exceeding the 240 mg/dl from week 8 to end of week 13<br>- any fasting plasma glucose exceeding the 200 mg/dl from week 14 to end of treatment

Table A.2. Definition of hypoglycemia.

Study name (identifier)	Criteria
AWARD-10 (NCT02597049)	Plasma glucose ≤ 70 mg/dl
DUAL IX (NCT02773368)	Either severe (requiring the assistance of another person) or blood glucose-confirmed symptomatic events (plasma glucose level <56 mg/dl with symptoms consistent with hypoglycemia)
DURATION-8 (NCT02229396)	Plasma glucose ≤ 70 mg/dl
SUSTAIN 9 (NCT03086330)	Plasma glucose ≤ 70 mg/dl

Figure A.1. Risk of bias graph: review of authors' judgements about each risk of bias item presented as percentages across all included studies.

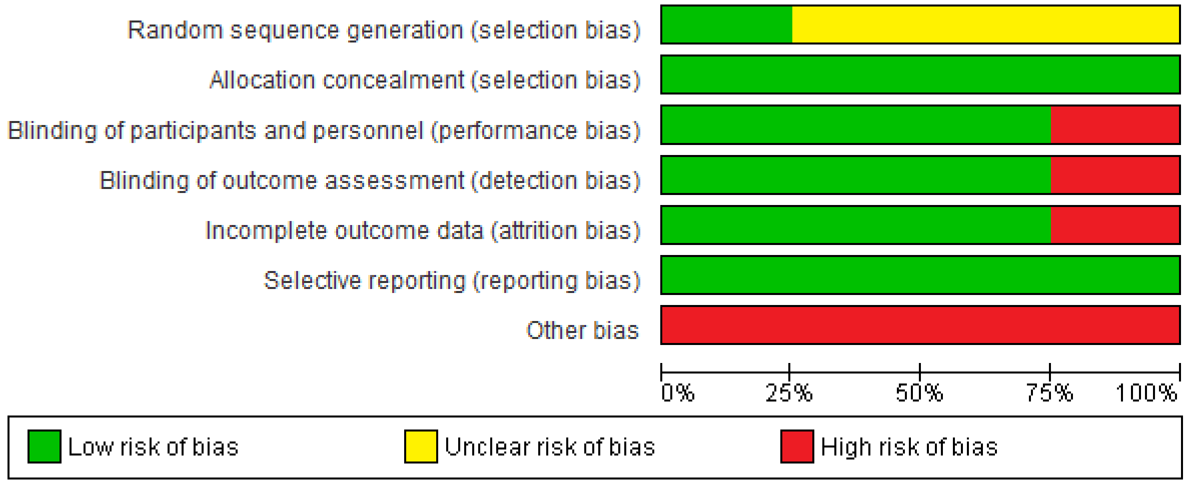

Figure A.2. Risk of bias summary: review of authors' judgements about each risk of bias item for each included study.

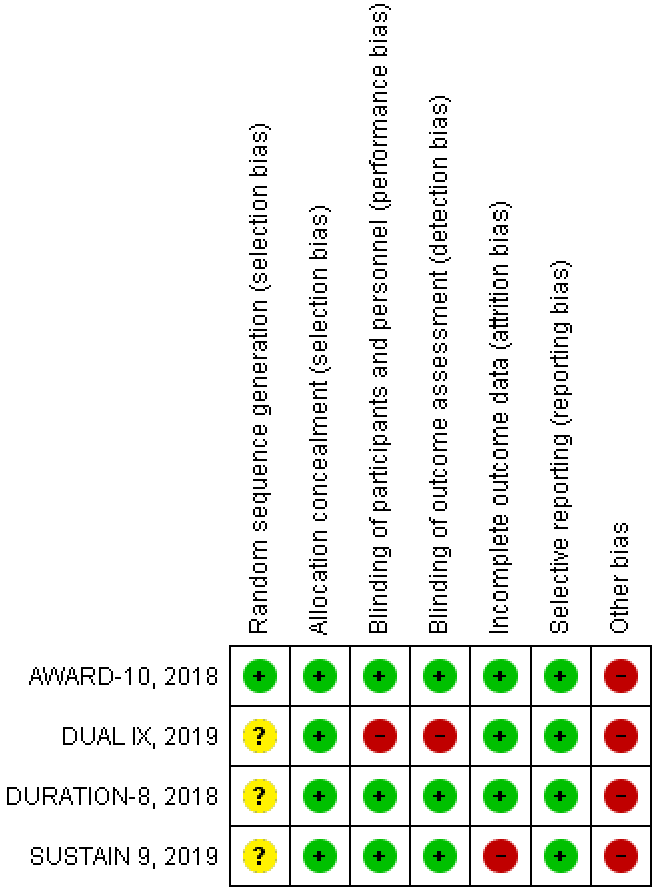

Figure A.3. Forest plot of meta-analysis for number of patients achieving HbA1c < 7%.

Study or Subgroup	Weight	Risk Ratio M-H, Random, 95% CI
AWARD-10, 2018 - Dulaglutide 0.75 mg	19.6%	1.92 [1.33, 2.77]
AWARD-10, 2018 - Dulaglutide 1.5 mg	19.7%	2.15 [1.52, 3.06]
DUAL IX, 2018	21.1%	1.19 [1.07, 1.32]
DURATION-8, 2018	19.8%	2.28 [1.63, 3.19]
SUSTAIN 9, 2019	19.8%	4.25 [3.01, 6.00]
**Total (95% CI)**	**100.0%**	**2.15 [1.20, 3.86]**
Total events		

Heterogeneity: Tau² = 0.42; Chi² = 92.43, df = 4 (P < 0.00001); I² = 96%
Test for overall effect: Z = 2.57 (P = 0.01)

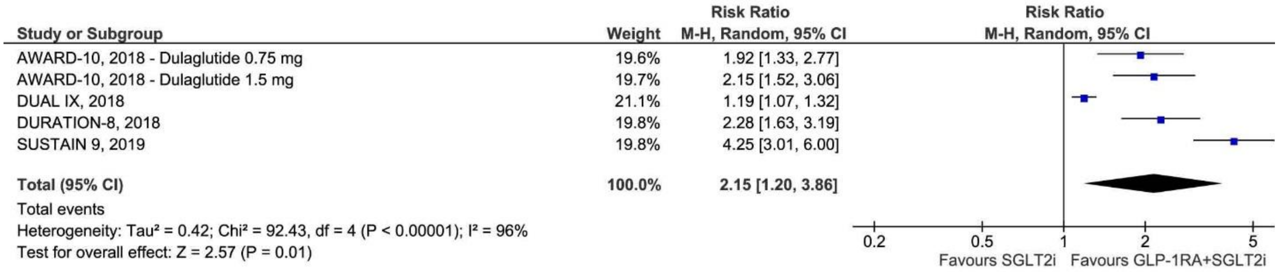

Figure A.4. Forest plot of meta-analysis for number of patients requiring rescue therapy due to hyperglycemia.

Study or Subgroup	Weight	Risk Ratio M-H, Random, 95% CI
AWARD-10, 2018 - Dulaglutide 0.75 mg	21.7%	0.30 [0.07, 1.21]
AWARD-10, 2018 - Dulaglutide 1.5 mg	18.3%	0.20 [0.04, 0.99]
DURATION-8, 2018	46.9%	0.71 [0.54, 0.93]
SUSTAIN 9, 2019	13.1%	0.13 [0.02, 0.99]
**Total (95% CI)**	**100.0%**	**0.37 [0.15, 0.89]**
Total events		

Heterogeneity: Tau² = 0.40; Chi² = 6.32, df = 3 (P = 0.10); I² = 53%
Test for overall effect: Z = 2.23 (P = 0.03)

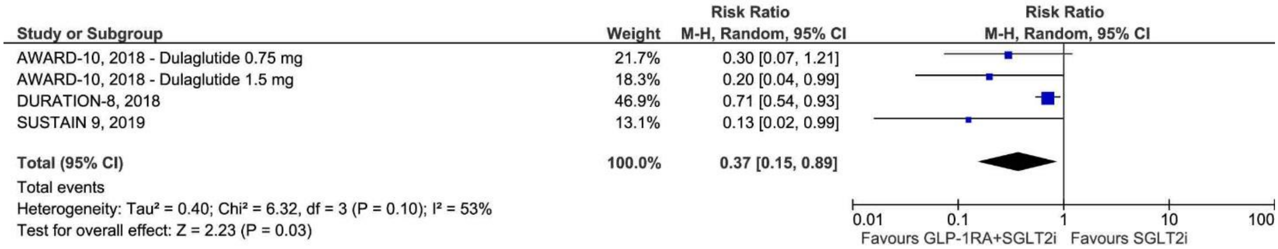

Figure A.5. Forest plot of meta-analysis for incidence of hypoglycemia.

Study or Subgroup	Weight	Risk Ratio IV, Random, 95% CI
AWARD-10, 2018 - Dulaglutide 0.75 mg	25.0%	1.23 [0.61, 2.50]
AWARD-10, 2018 - Dulaglutide 1.5 mg	25.1%	1.48 [0.74, 2.93]
DUAL IX, 2018	27.4%	0.41 [0.34, 0.49]
SUSTAIN 9, 2019	22.5%	7.54 [2.67, 21.30]
**Total (95% CI)**	**100.0%**	**1.43 [0.46, 4.52]**

Heterogeneity: Tau² = 1.24; Chi² = 46.85, df = 3 (P < 0.00001); I² = 94%
Test for overall effect: Z = 0.62 (P = 0.54)

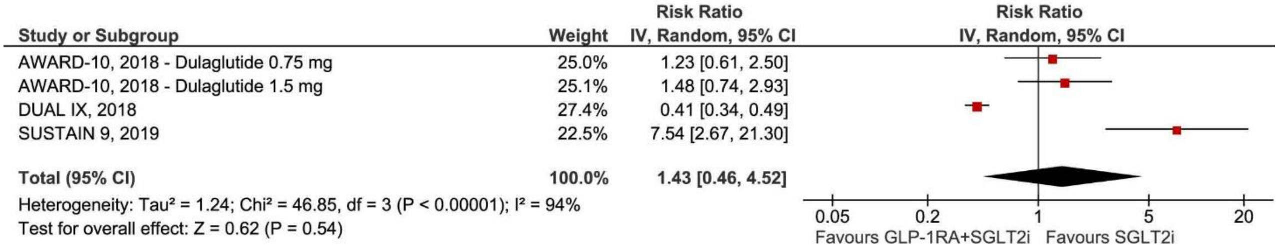

Figure A.6. Forest plot of meta-analysis for change in Total Cholesterol from baseline to the last available follow-up.

Study or Subgroup	Weight	Mean Difference IV, Random, 95% CI
AWARD-10, 2018 - Dulaglutide 0.75 mg	22.6%	-0.10 [-0.26, 0.06]
AWARD-10, 2018 - Dulaglutide 1.5 mg	22.5%	-0.10 [-0.26, 0.06]
DUAL IX, 2018	17.7%	-0.15 [-0.38, 0.08]
DURATION-8, 2018	18.4%	-0.04 [-0.26, 0.18]
SUSTAIN 9, 2019	18.8%	-0.50 [-0.71, -0.29]
**Total (95% CI)**	**100.0%**	**-0.17 [-0.32, -0.02]**

Heterogeneity: Tau² = 0.02; Chi² = 11.82, df = 4 (P = 0.02); I² = 66%
Test for overall effect: Z = 2.26 (P = 0.02)

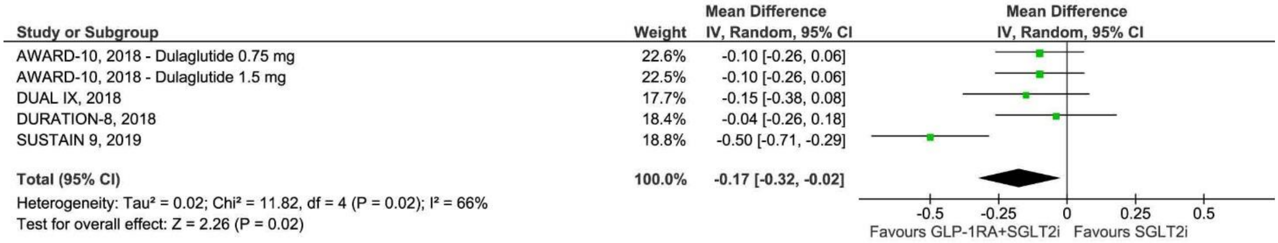

Figure A.7. Forest plot of meta-analysis for change in HDL Cholesterol from baseline to the last available follow-up.

Study or Subgroup	Weight	Mean Difference IV, Random, 95% CI
AWARD-10, 2018 - Dulaglutide 0.75 mg	20.7%	0.01 [-0.04, 0.06]
AWARD-10, 2018 - Dulaglutide 1.5 mg	22.7%	0.03 [-0.02, 0.08]
DUAL IX, 2018	15.3%	-0.02 [-0.08, 0.04]
DURATION-8, 2018	16.6%	0.00 [-0.05, 0.05]
SUSTAIN 9, 2019	24.7%	0.00 [-0.05, 0.05]
**Total (95% CI)**	**100.0%**	**0.01 [-0.02, 0.03]**

Heterogeneity: Tau² = 0.00; Chi² = 1.93, df = 4 (P = 0.75); I² = 0%
Test for overall effect: Z = 0.51 (P = 0.61)

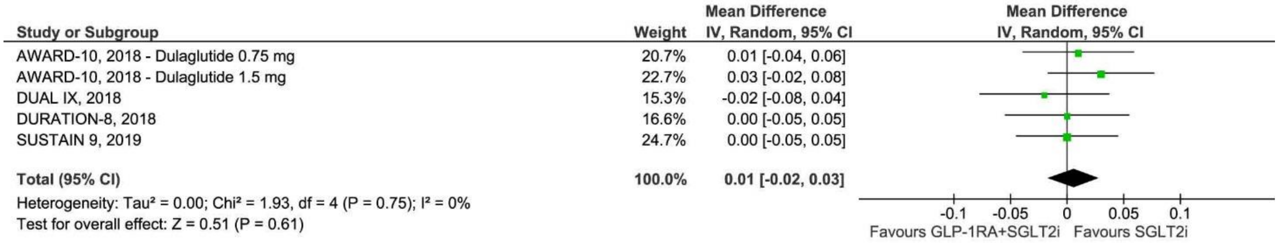

Figure A.8. Forest plot of meta-analysis for change in LDL Cholesterol from baseline to the last available follow-up.

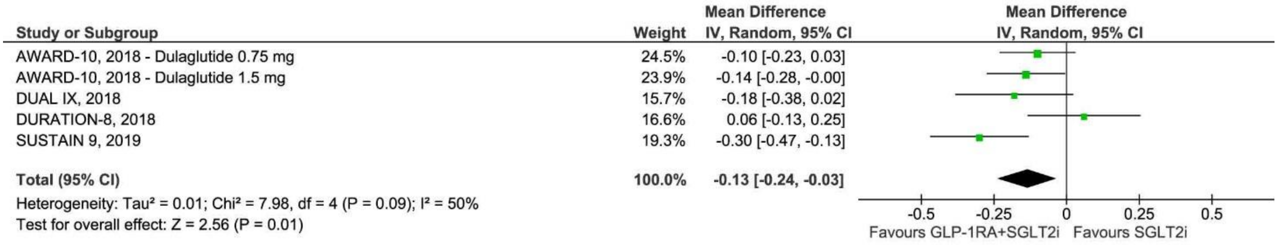

Figure A.9. Forest plot of meta-analysis for change in Triglycerides from baseline to the last available follow-up.

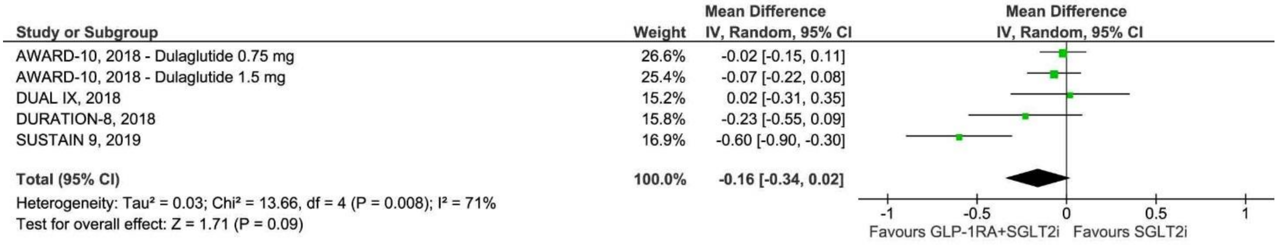

Table A.3. Sensitivity analysis and publication bias.

Study name (identifier)	HbA1c	Body weight	Systolic blood pressure	Total cholesterol	HDL Cholesterol	LDL Cholesterol	Triglycerides	HbA1c<7%	Rescue therapy	Hypoglycemic events
AWARD-10 (NCT02597049) - Dulaglutide 0.75 mg	**0.005**	**0.004**	**<0.001**	**0.05**	0.71	**0.04**	0.10	**0.03**	0.08	0.58
AWARD-10 (NCT02597049) - Dulaglutide 1.5 mg	**0.008**	**0.01**	**0.001**	**0.05**	0.92	0.06	0.16	**0.04**	0.08	0.63
DUAL IX (NCT02773368)	**<0.001**	0.08	**0.004**	0.06	0.40	**0.05**	0.07	**<0.001**	-	0.10
DURATION-8 (NCT02229396)	**0.001**	**0.02**	**<0.001**	**0.02**	0.58	**<0.001**	0.17	**0.04**	**0.001**	-
SUSTAIN 9 (NCT03086330)	**<0.001**	**0.006**	**<0.001**	**0.04**	0.56	**0.03**	0.26	**0.01**	0.06	0.77
Egger's test	0.442	0.694	0.821	0.553	0.283	0.951	0.300	**0.042**	**0.005**	**0.022**
